# Accurate and efficient data acquisition methods for high-resolution angle-resolved photoemission microscopy

**DOI:** 10.1038/s41598-018-34894-7

**Published:** 2018-11-27

**Authors:** Hideaki Iwasawa, Hitoshi Takita, Kazuki Goto, Wumiti Mansuer, Takeo Miyashita, Eike F. Schwier, Akihiro Ino, Kenya Shimada, Yoshihiro Aiura

**Affiliations:** 1 Hiroshima Synchrotron Radiation Center, Hiroshima University, Higashi-Hiroshima, Hiroshima 739-0046 Japan; 2 Diamond Light Source, Harwell Science and Innovation Campus, Didcot, OX11 0DE UK; 3 Graduate School of Science, Hiroshima University, Higashi-Hiroshima, Hiroshima 739-8526 Japan; 4 Department of Education and Creation Engineering, Kurume Institute of Technology, Kurume, Fukuoka 830-0052 Japan; 5 National Institute of Advanced Industrial Science and Technology, Ibaraki, 305-8568 Japan

## Abstract

Angle-resolved photoemission spectroscopy (ARPES) is a powerful experimental technique in materials science, as it can directly probe electronic states inside solids in energy (*E*) and momentum (*k*) space. As an advanced technique, spatially-resolved ARPES using a well-focused light source (high-resolution ARPES microscopy) has recently attracted growing interests because of its capability to obtain local electronic information at micro- or nano-metric length scales. However, there exist several technical challenges to guarantee high precision in determining translational and rotational positions in reasonable measurement time. Here we present two methods of obtaining *k*-space mapping and real-space imaging in high-resolution ARPES microscopy. One method is for *k*-space mapping measurements that enables us to keep a target position on a sample surface during sample rotation by compensating rotation-induced displacements (tracing acquisition method). Another method is for real-space imaging measurements that significantly reduces total acquisition time (scanning acquisition method). We provide several examples of these methods that clearly indicate higher accuracy in *k*-space mapping as well as higher efficiency in real-space imaging, and thus improved throughput of high-resolution APRES microscopy.

## Introduction

Understanding of local electronic structures at small length scales is essential to improve designing and engineering of micro- and nanoscale devices. This leads to a growing demand to advance the characterization tools with a matched length-scale. Angle-resolved photoemission spectroscopy (ARPES) has been a promising probe of micro/nano-scopic electronic states by focusing an incident beam (high-resolution ARPES microscopy)^1–4^. However, there exist several technical challenges to preserve its resolving power in *k*- and real-spaces, which require novel developments on data acquisition method to realize accurate and efficient high-resolution ARPES microscopy.

In ARPES experiments, energy distributions of electrons are measured as a function of emission angles of photoelectrons from single crystals. Conventionally, to map out the emission-angle distribution of photoelectrons, the sample holder or the electron analyzer have to be rotated *in situ* (there is an alternative way to map out the emission-angle distribution of photoelectrons by using a deflector-type electron analyzer that has been recently developed). In many modern ARPES systems^5,6^, a cryogenic sample manipulator compatible with an ultrahigh vacuum and low temperature is integrated to control sample orientation (polar/tilt/azimuthal)^6,7^. However, sample rotations inevitably produce a displacement of a detection position on the sample surface because the rotational center of the sample manipulator is not placed on the detection position in general. Therefore, the smaller spot size as well as the region-of-interest becomes, the more problematic such displacement is in high-resolution ARPES microscopy experiments.

Another important issue is that high-resolution ARPES microscopy is a time-consuming experiment since the high spatial-resolution increases the sensitivity to local quality variations on the sample surface. Therefore, spatial ARPES imaging of the sample surface has been often performed by a sequential and point-by-point acquisition. In this imaging-mode, the total acquisition time is proportional to the number of data points, which in turn are a sum of the main ARPES acquisition time and overhead-time comprised of mechanical translational and angular adjustments and those stabilizations as well as the data file creation. It is obvious that the overhead-time becomes a bottleneck for ARPES imaging, rather than the necessary ARPES acquisition time itself.

In this study, we developed two kinds of data acquisition methods focusing on the accurate *k*-space mapping and efficient real-space imaging in high-resolution ARPES microscopy experiments. The former method was designed to compensate the rotational displacements during *k*-space mapping by means of a translational correction computed through three dimensional rotational matrices. This allows us to control sample orientations (polar, tilt, and/or azimuthal angles of the sample) while guaranteeing a constant measurement position on the sample surface. In the latter method, we upgraded the imaging-mode with a scanning-type acquisition instead of a sequential point-by-point ARPES data acquisition for real-space imaging. This enabled us to significantly shorten the time-consuming spatial ARPES imaging measurements retaining local topographic and electronic information. We demonstrate that these methods significantly improve the accuracy, efficiency, and throughput in high-resolution ARPES microscopy experiments.

## Results and Discussions

### Overview of high-resolution ARPES microscopy system

**Figure 1:**
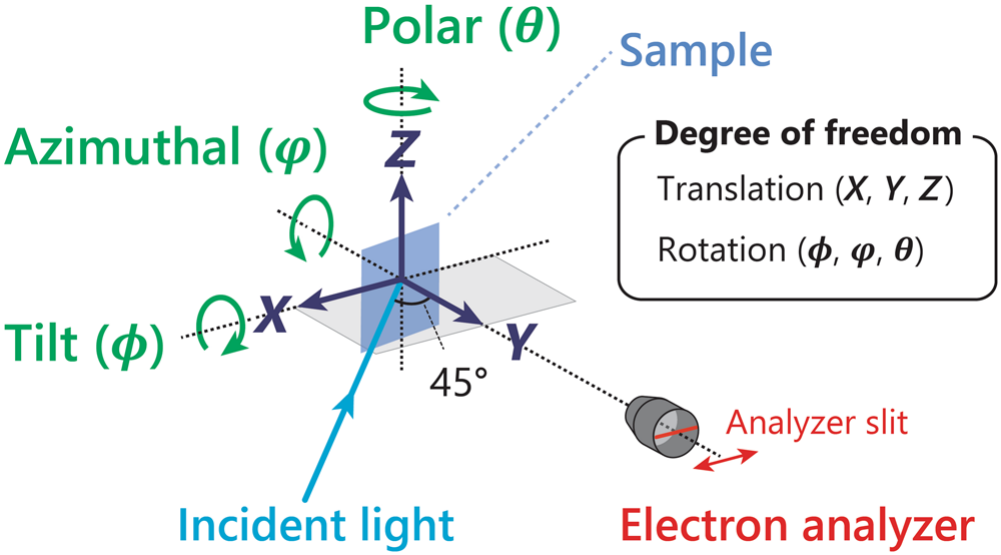
Experimental geometry for the present μ-ARPES measurement where sample rotational and translational degrees of freedom are manipulated by high-precision sample goniometer and stage, respectively.

Figure 1 shows an experimental setup for high-resolution ARPES microscopy (μ-ARPES) system developed at the Hiroshima Synchrotron Radiation Center (HiSOR) as described in Methods and elsewhere^4^. The present μ-ARPES system achieves a micron-order spatial resolution besides ultimate energy and momentum resolutions (*ΔE* < 0.26 meV, *Δk* < 6 × 10^−4^ Å^−1^)^4^. The micro-focused laser light incidents at an angle of 45° from the focal direction of a high-resolution hemispherical electron analyzer (R4000, Scienta Omicron), and the analyzer slit lies on the incidence plane of the laser light. The translational axes are defined as that the *X*-axis and *Z*-axis are parallel and perpendicular to the analyzer slit, respectively, and the *Y*-axis is along the lens axis of the analyzer. Then, the rotational axes are defined against each translational axis as the polar (*ϑ*), tilt (*φ*), and azimuthal (*ϕ*) angles of the rotation around the *Z*-axis, *X*-axis, and *Y*-axis.

The translational degrees of freedom (*X*, *Y*, *Z*) of the sample are manipulated by a high-precision *XYZ* translator stage (iXYZ, the ExPP Co. Ltd.) with high enough accuracy for μ-ARPES. The rotational degrees of freedom (*ϕ*, *φ*, *ϑ*) of the sample are provided by a (*ϕ*, *φ*)-type sample goniometer with a liquid-helium-flow cryostat (i-GONIO LT, R-Dec Co. Ltd.)^7^ and a high precision rotary stage (iRS152, Vacuum and Optical Instruments Co. Ltd.) for *ϑ* angle^8^. Note that, with the present manipulator, the *XYZ*-motion and (*ϕ*, *ϑ*) rotation are driven by a computer-controlled stepping motor, while the remaining *φ*-rotation is adjusted manually by using a screw jointed to the goniometer by a wobble stick. All the motor motions are controlled via home-made software coded by using LabVIEW (National Instruments), which was further developed to link with ARPES data acquisition programs (SESWrapper, Scienta Omicron). With this setup, we can automatically acquire ARPES data sets for *k*-space ARPES mapping by changing the *ϕ*-angle as well as spatial imaging of the sample surface by scanning the *X*-axis and/or *Z*-axis. In the following, we present concepts of two data acquisition methods for (1) *k*-space ARPES mapping and (2) spatial ARPES imaging, and evaluations of these methods via representative μ-ARPES experiments.

### Concept of tracing ARPES acquisition method

**Figure 2:**
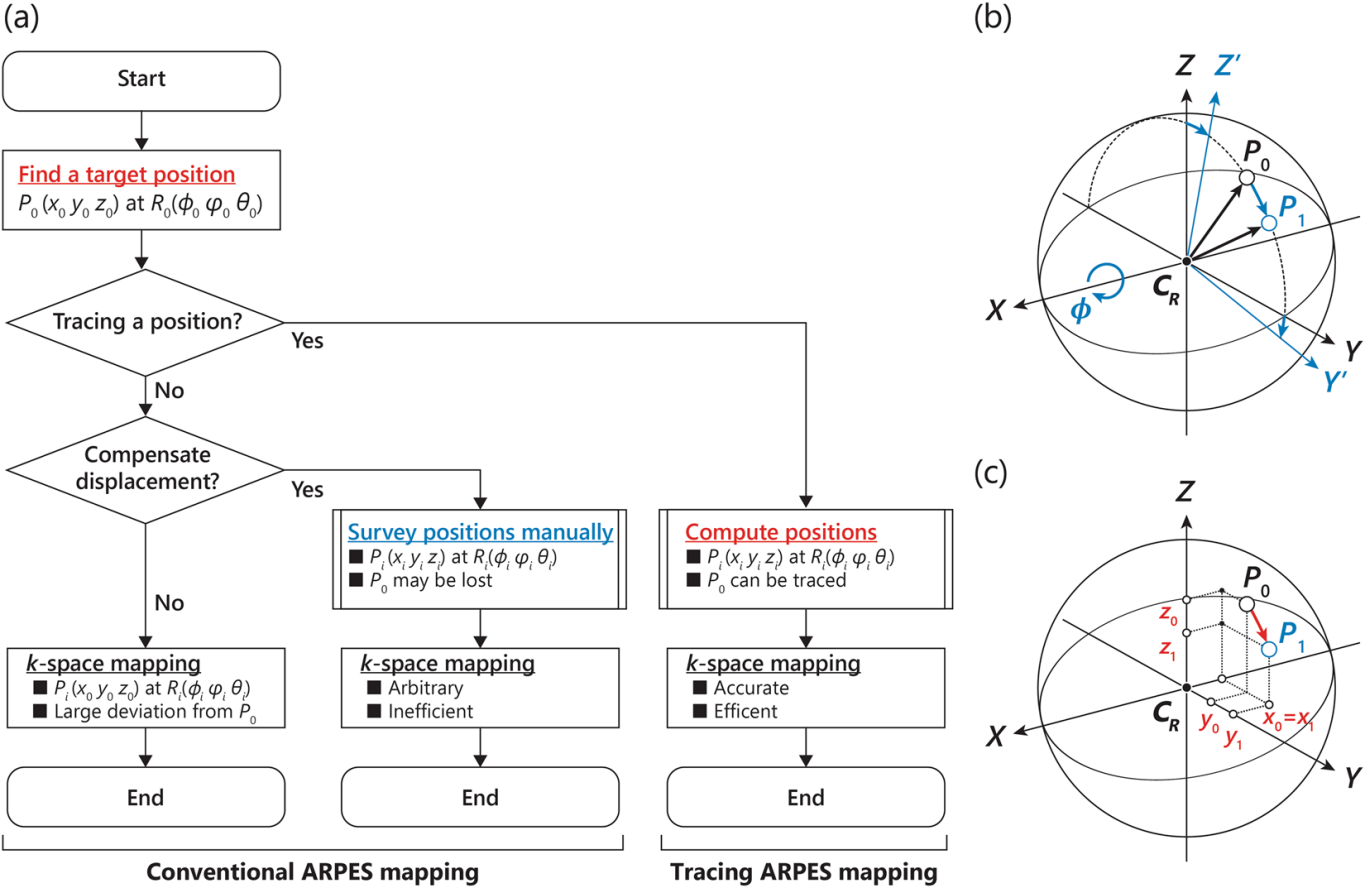
Rotational displacements and the concept of computational compensation. (**a**) Flow chart comparing (left and middle) conventional ARPES mappings and (right) tracing ARPES mapping. (Left) Starting from a position of *P*_0_(*x*_0_, *y*_0_, *z*_0_) and angle of *R*_0_(*ϕ*_0_, *φ*_0_, *ϑ*_0_), ARPES mapping is performed by varying only angles to *R*_*i*_(*ϕ*_*i*_, *φ*_*i*_, *ϑ*_*i*_) without any compensations, where the index *i* represents a number of 0, 1, …, n. (Middle) ARPES mappings are performed at several different positions *P*_*i*_(*x*_*i*_, *y*_*i*_, *z*_*i*_) at each of angles *R*_*i*_(*ϕ*_*i*_, *φ*_*i*_, *ϑ*_*i*_) based on the manual survey to trace the original position *P*_0_(*x*_0_, *y*_0_, *z*_0_) at angles *R*_0_(*ϕ*_0_, *φ*_0_, *ϑ*_0_), which may or may not reduce the rotational displacements but surely requires a certain time. (Right) Tracing ARPES measurements in which the target positions *P*_i_(*x*_i_, *y*_i_, *z*_i_) at any angles *R*_i_(*ϕ*_i_, *φ*_i_, *ϑ*_i_) can be computed from the original position *P*_0_(*x*_0_, *y*_0_, *z*_0_) at angles *R*_0_(*ϕ*_0_, *φ*_0_, *ϑ*_0_) without ambiguity. (**b)** Schematic drawing of a tilt (*ϕ*)-rotation along the *X* axis, and **c** the associated rotational displacements appeared in the coordinates from *P*_0_(*x*_0_, *y*_0_, *z*_0_) to *P*_1_(*x*_1_, *y*_1_, *z*_1_) except for *x*_0_ = *x*_1_.

We discuss the *k*-space ARPES mapping by varying sample orientations and accompanied rotational displacements that should be corrected for high-resolution ARPES microscopy experiments. Figure 2a summarizes the flow of the conventional ARPES mapping and the tracing ARPES mapping by sample rotations to obtain electronic states in *k*-space; (left) APRES mapping is performed without any compensations by varying the angles *R*_*i*_ (*ϕ*_*i*_, *φ*_*i*_, *ϑ*_*i*_) starting from an original position *P*_0_ (*x*_0_, *y*_0_, *z*_0_) at angles *R*_0_ (*ϕ*_0_, *φ*_0_, *ϑ*_0_), inevitably leading to the rotational displacements in real space unless the original position *P*_0_ is located at the center of rotation *C*_R_ (*x*_CR_, *y*_CR_, *z*_CR_). (Middle) APRES mapping is performed based on the manual survey of plausible positions *P*_*i*_ (*x*_*i*_, *y*_*i*_, *z*_*i*_) at some of the angles *R*_*i*_ (*ϕ*_*i*_, *φ*_*i*_, *ϑ*_*i*_) where *i* represents different measurement steps, and a simple interpolation (linear, polynomial, trigonometric, etc.) is used based on the obtained set of positions. Sometimes this method works well, but sometimes fails to trace the original position on the sample after spending a certain time to find optimized positions. More importantly, these conventional ARPES mapping methods are arbitrary as manual judgement of the position is required at each sample position. (Right) Alternatively, we developed a more accurate and efficient method to trace the original position by computing the target positions *P*_*i*_ (*x*_*i*_, *y*_*i*_, *z*_*i*_) at any angles *R*_*i*_ (*ϕ*_*i*_, *φ*_*i*_, *ϑ*_*i*_) without ambiguity. In the following, we will elaborate on this ‘tracing ARPES mapping method’ to improve accuracy of high-resolution ARPES microscopy experiments.

Figure 2b illustrates an example of the rotational displacements due to the tilt rotation by an angle of *ϕ* with respect to the center of rotation *C*_R_ (*x*_CR_, *y*_CR,_ *z*_CR_). The coordinates are varied from *P*_0_ (*x*_0_, *y*_0_, *z*_0_) (original positions) to *P*_1_ (*x*_1_, *y*_1_, *z*_1_) after the sample rotation as shown in Fig. 2c. The rotational displacements are described by $\Delta P=\,\overrightarrow{O{P}_{1}}-\overrightarrow{O{P}_{0}}=(0,\,{y}_{1}-{y}_{0},\,{z}_{1}-{z}_{0})$ as the rotation around the *X*-axis should not change the *X*-coordinate (*x*_0_ = *x*_1_). To evaluate such displacements quantitatively, we consider that any three dimensional rotations with respect to the center of rotation can be expressed by a 4-by-4 determinant. Assuming the rotation of *R* (*ϕ*, *φ*, *ϑ*) with respect to the center of rotation *C*_R_ (*x*_CR_, *y*_CR_, *z*_CR_), the original coordinates *P*_0_ (*x*_0_, *y*_0_, *z*_0_) are transformed into the new coordinates *P*_1_ (*x*_1_, *y*_1_, *z*_1_) as follows;

1 $$(\begin{array}{c}{x}_{1}\\ {y}_{1}\\ {z}_{1}\\ 1\end{array})={T}_{{\rm{CR}}}^{+}{R}_{Z}({\vartheta }){R}_{Y}(\phi ){R}_{X}(\varphi ){T}_{{\rm{CR}}}^{-}(\begin{array}{c}{x}_{0}\\ {y}_{0}\\ {z}_{0}\\ 1\end{array}),$$

where ${T}_{{\rm{CR}}}^{\pm }$ are the translational matrices given by

$${T}_{{\rm{CR}}}^{\pm }=(\begin{array}{cccc}1 & 0 & 0 & \pm {x}_{{\rm{CR}}}\\ 0 & 1 & 0 & \pm {y}_{{\rm{CR}}}\\ 0 & 0 & 1 & \pm {z}_{{\rm{CR}}}\\ 0 & 0 & 0 & 1\end{array}),$$

and ${R}_{X}(\varphi )$, ${R}_{Y}(\phi )$, ${R}_{Z}({\vartheta })$ are the rotational matrices with respect to the *X*, *Y*, *Z*-axis given by

$$\begin{array}{c}{R}_{X}(\varphi )=(\begin{array}{cccc}1 & 0 & 0 & 0\\ 0 & \cos \,\varphi  & \sin \,\varphi  & 0\\ 0 & -\sin \,\varphi  & \cos \,\varphi  & 0\\ 0 & 0 & 0 & 1\end{array}),\,{R}_{Y}(\phi )=(\begin{array}{cccc}\cos \,\phi  & 0 & -\sin \,\phi  & 0\\ 0 & 1 & 0 & 0\\ \sin \,\phi  & 0 & \cos \,\phi  & 0\\ 0 & 0 & 0 & 1\end{array}),\\ \,{R}_{Z}({\vartheta })=(\begin{array}{cccc}\cos \,{\vartheta } & \sin \,{\vartheta } & 0 & 0\\ -\sin \,{\vartheta } & \cos \,{\vartheta } & 0 & 0\\ 0 & 0 & 1 & 0\\ 0 & 0 & 0 & 1\end{array}).\end{array}$$

As a practical example, we calculate the rotational displacements due to one-dimensional tilt-rotation along the *X*-axis by *ϕ* = ±10°. Assuming that the original position *P*_0_(*x*_0_, *y*_0_, *z*_0_) is offset by 1 mm (a typical sample size) along the *Y*-axis (*Z*-axis) from the center of rotation (*y*_0_ = *y*_CR_ ± 1 mm or *z*_0_ = *z*_CR_ ± 1 mm), the *ϕ* = ±10° rotation leads to the rotational displacements by ±15 μm in the *Y*-axis (*Z*-axis) and ±174 μm in the *Z*-axis (*Y*-axis). Importantly, the rotational displacements are increased with the degrees of rotation and the deviation from the center of rotation, and they are not negligible in most high-resolution ARPES microscopy experiments.

At the same time, however, Eq. 1 also means new coordinates *P*_*i*_ (*x*_*i*_, *y*_*i*_, *z*_*i*_) can be calculated and hence traceable for any angles *R*_*i*_ (*ϕ*_*i*_, *φ*_*i*_, *ϑ*_*i*_) based on the target coordinates *P*_0_ (*x*_0_, *y*_0_, *z*_0_) if the center of rotation *C*_R_ (*x*_CR_, *y*_CR_, *z*_CR_) is known. As described in the Supplementary material, the center of rotation *C*_R_ (*x*_CR_, *y*_CR_, *z*_CR_) has been determined with a 10-μm accuracy based on optical microscope images capturing a laser spot on a phosphor reference sample (Fig. S1). The following evaluation of the tracing ARPES method has been carried out based on the determined center of rotation *C*_R_ (*x*_CR_, *y*_CR_, *z*_CR_). It should be commented here that the rotational axes actually do not meet at a specific point in our μ-ARPES system, and hence, Eq. 1 requires slight modifications in a practical usage as detailed in the supplementary material.

### Evaluation of tracing ARPES acquisition method

**Figure 3:**
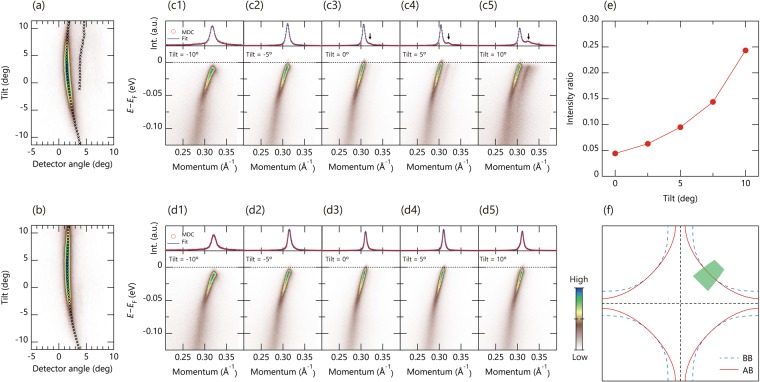
Evaluation of tracing ARPES method via μ-ARPES measurements on a high-*T*_c_ superconducting cuprate Bi_2_Sr_2_CaCu_2_O_8+δ_ taken with a photon energy of 6.43 eV and a temperature below 20 K near the nodal direction. (**a**,**b)** Fermi surface mapping obtained by tilt rotations (**a**) without and (**b**) with the tracing ARPES method. Here, the axis scales are in raw angles for simplicity in comparison between two measurement methods. (c1–c5, d1–d5), ARPES image plots together with the momentum distribution curve at the Fermi level measured (c1–c5) without and (d1–d5) with the computational tracing at several tilt angles as indicated in the figures. (**e)** Relative intensity plot of the ghost structure against the main structure as indicated by an arrow in (c3–c5) as a function of the tilt angle, where error bars are smaller than the markers and negligible. (**f)** Schematic Fermi surface plots for the bonding and antibonding CuO_2_-plane bands (BB and AB), where the green shaded area represent the measureable position when the tilt angles are given by ±10 deg with respect to the (0, 0)-(π, π) nodal direction. Note that present measurements are not exactly corresponding to the region indicated in (**f**) as the nodal direction is placed around a tilt angle of +5 deg in the results of tracing ARPES method.

We examine the capabilities of the tracing ARPES method via several Fermi surface mappings of a high-*T*_c_ superconducting cuprate Bi_2_Sr_2_CaCu_2_O_8+δ_ (Bi2212) single crystal taken with a photon energy of 6.43 eV at a temperature below 20 K. To highlight effects of rotational displacements, we have selected the original measurement position at which multiple domains exist in adjacent real spaces on the sample surface. The Fermi surface of Bi2212 consists of the bilayer-split antibonding and bonding bands of the CuO_2_ planes (AB and BB) as shown in Fig. 3f. Whereas, the low-excitation energy used here allows to excite the AB only^9^, providing a simple situation suitable for the present purpose.

Figure 3a,b show the Fermi surface of Bi2212 measured by the conventional and tracing ARPES methods near the (0, 0)-(π, π) nodal region as indicated by the green shaded area in Fig. 3f. Both mappings start from the position of *P*_0_ (*x*_0_, *y*_0_, *z*_0_) and the angle of *R*_0_ (−10, 0, 40) and measured from negative to positive tilt angles. While the tilt angles were only varied in the conventional mapping, sample positions *P*_*i*_ (*x*_*i*_, *y*_*i*_, *z*_*i*_) were also adjusted at each tilt-angle of *R*_*i*_ (*ϕ*_*i*_, 0, 40) based on the computations of Eq. 1 in the tracing mapping. Corresponding ARPES images measured by the conventional and tracing methods are shown in Fig. 3c1–5 and 3d1–5, respectively, together with the momentum distribution curves (MDCs) at the Fermi level (upper panels). It is obvious that a weak but distinct ghost structure appears in the right-side nearby the main AB (arrow in MDCs of Fig. 3c3–5) in case of the conventional ARPES method. On the other hand, such ghost structure is completely absent for the tracing ARPES method. It should be noted that the observed ghost intensity is different from the BB of Bi2212 because the observed splitting is too large compared with the reported bilayer splitting (0.015 Å^−1^)^10^, and the position is not symmetric with respect to the nodal direction as seen in the peak positions of MDCs at the Fermi level indicated by the markers in Fig. 3a. It is thus reasonable to attribute the observed ghost structure to a different surface domain due to the rotational displacements. Indeed, the relative intensity of the ghost structure against the main AB becomes larger from ~5% (*ϕ* = 0°) to ~25% (*ϕ* = +10°) as shown in Fig. 3e, consistent with the natural expectation that the rotational displacements become larger when being farther away from the starting angle. Consequently, all these results demonstrated that the present tracing APRES method enables us to track a target measurement position on the sample during rotation of the sample, and to obtain reliable and intrinsic local information by high-resolution ARPES microscopy experiments.

### Concept of scanning ARPES acquisition method

High-resolution ARPES microscopy is a time-consuming experiment as it requires mapping out the large number of data points in the real space. It is therefore significantly important to speed up the real-space ARPES imaging measurements as much as possible while retaining their qualitative information. Here we examine two methods denoted here as stepping and scanning ARPES acquisition methods. Note that in this section the sample position means not only the spatial coordinates but also angle orientations, namely, *P* (*x*, *y*, *z, ϕ*, *φ*, *ϑ*).

**Figure 4:**
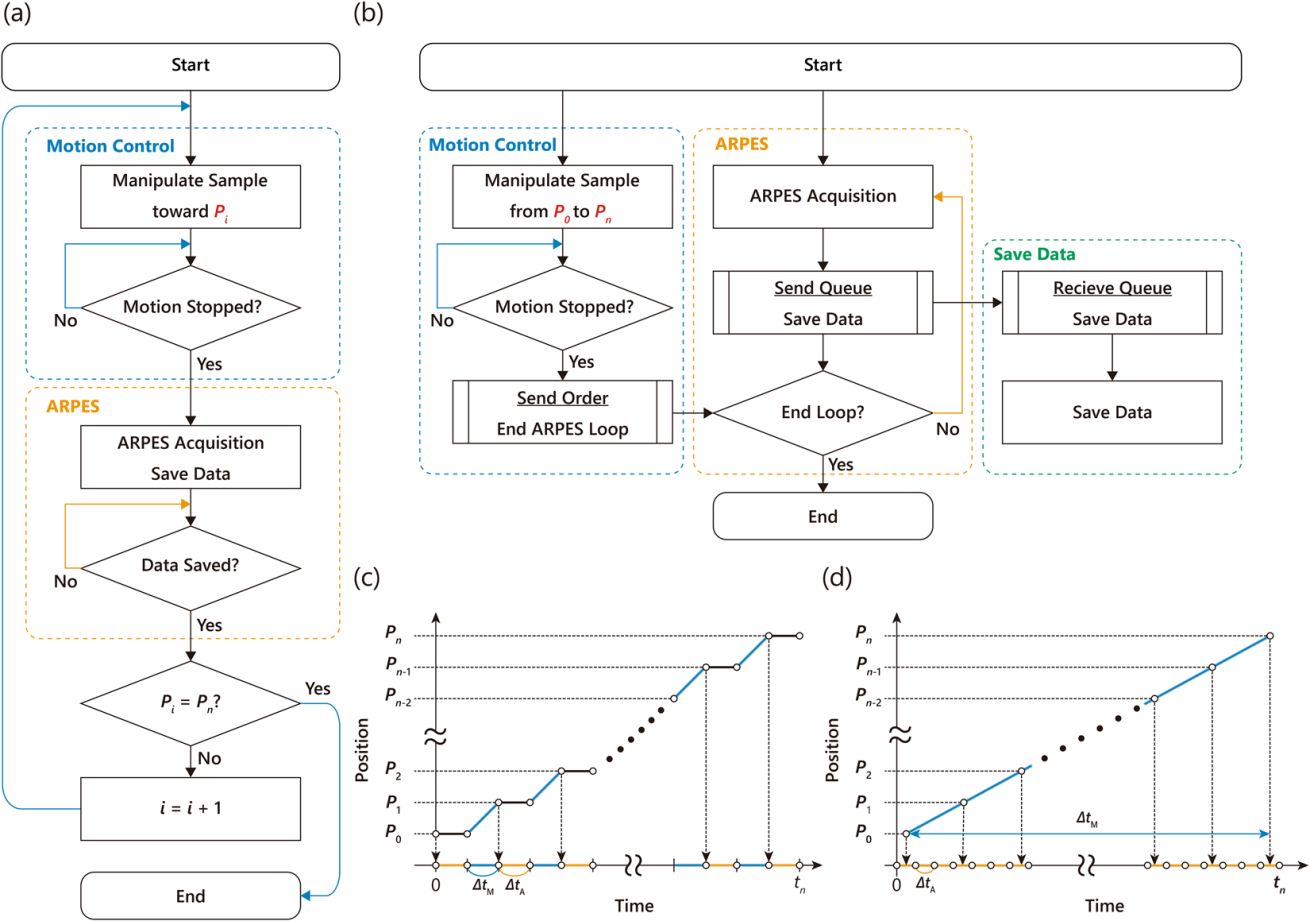
Concept of ARPES-imaging; conventional ‘stepping’ acquisition vs. ‘scanning’ acquisition. (**a**) Flow chart of ARPES-imaging with the conventional step-by-step acquisition. The mapping consists of sequential iterations in which a motion control and ARPES acquisition are performed step-by-step in this order until a current position *P*_*i*_ arrives a destination *P*_*n*_ by proceeding sequentially (*i* = *i* + 1) from a starting point *P*_0_ while *i* < *n* (*i* = 0, 1, 2…). (**b**) Flow chart of ARPES-imaging with the scanning acquisition. The mapping consists of three almost independent sections that are processing in parallel. Motion control and ARPES acquisition sections starts simultaneously; In the motion control section, positions and/or orientations of the samples are changed as a single action from a starting point *P*_0_ to a destination *P*_*n*_. When the motion stopped, an order is sent to the ARPES acquisition section to end all the mapping acquisition. In the ARPES acquisition section, ARPES acquisition runs continuously in a loop until the ending order received. The ARPRES acquisition section also sends a queue order and dataset to a section of data saving after each ARPES acquisition. (**c,d)** Schematic diagram of sample position as a function of time for the conventional and scanning ARPES acquisition, respectively, where the motion control in one action takes a time by *Δt*_M_ (blue lines) and the ARPES acquisition takes a time by *Δt*_A_ (orange lines).

Figure 4a,b illustrate the concepts of stepping and scanning ARPES acquisition methods. As shown in Fig. 4a, the stepping ARPES mapping method consists of sequential iterations in which a motion control and an ARPES acquisition are performed step-by-step in this order. The iterations continue until a current position *P*_*i*_ arrives at a destination *P*_*n*_. In contrast, the scanning ARPES acquisition method shown in Fig. 4b divides measurement procedures into three parts; (1) Motion control, (2) ARPES acquisition, and (3) Save Data. Different from the stepping method, these three parts are almost independent and processed in parallel. In the motion control section, the position *P* (*x*, *y*, *z, ϕ*, *φ*, *ϑ*) is changed as a single action from a starting point *P*_0_ to a destination *P*_*n*_. When the motion stopped, a command is sent to the ARPES acquisition section in order to end all the acquisition. On the other hand, ARPES acquisition runs continuously in a loop until the stopping command received. This program sends a queue of orders to a data saving section after each ARPES acquisition. The measured data will be piled up and saved sequentially, which is independent from other two processes.

The differences between two acquisition modes can be seen more clearly in Fig. 4c,d, where a schematic diagram of sample position as a function of time is shown for the stepping and scanning ARPES acquisition methods, respectively. In both figures, the time needed for the motion control and ARPES acquisition for each step is indicated by *Δt*_M_ as blue lines and *Δt*_A_ as orange lines. In the stepping acquisition method, the motion control and ARPES acquisition are alternatingly repeated for each measurement step. In other words, the ARPES data acquisition can start only after that the motion control has been finished. Similarly, motion control can only be performed after the ARPES data have been acquired and saved. Hence, there inevitably exists significant overhead-time during these cycles, though such dead-time is not clearly indicated in Fig. 4c,d. This problem restricts a possible speed up of the stepping ARPES acquisition method because the total acquisition time is linearly proportional to the number of data points as each data point has a given overhead-time.

In contrast, in the scanning ARPES acquisition method, the two main processes of the motion control and ARPES acquisition can be operated in parallel, and the data saving process is separated from the others. This structure enables us to run continuous ARPES measurements without interruption. Although there exist a time-interval (dead time) in the process of ARPES acquisitions, such dead time is typically of the order of a couple 100 msec and can easily be countered in real live measurements by adjusting the speed and integration time of the scanning mode. Consequently, total acquisition time in the scanning ARPES acquisition method is almost identical to the time required for motion control in the case of one-dimensional ARPES mapping. We should note here that the scanning mode inevitably has slight ambiguity on the one-to-one correspondence between the ARPES spectrum and the coordinate information because the ARPES measurements are performed while moving the position. However, such errors can be practically negligible in the acquisition of the overview real-space imaging as we will show later.

### Evaluation of scanning ARPES acquisition method

**Figure 5:**
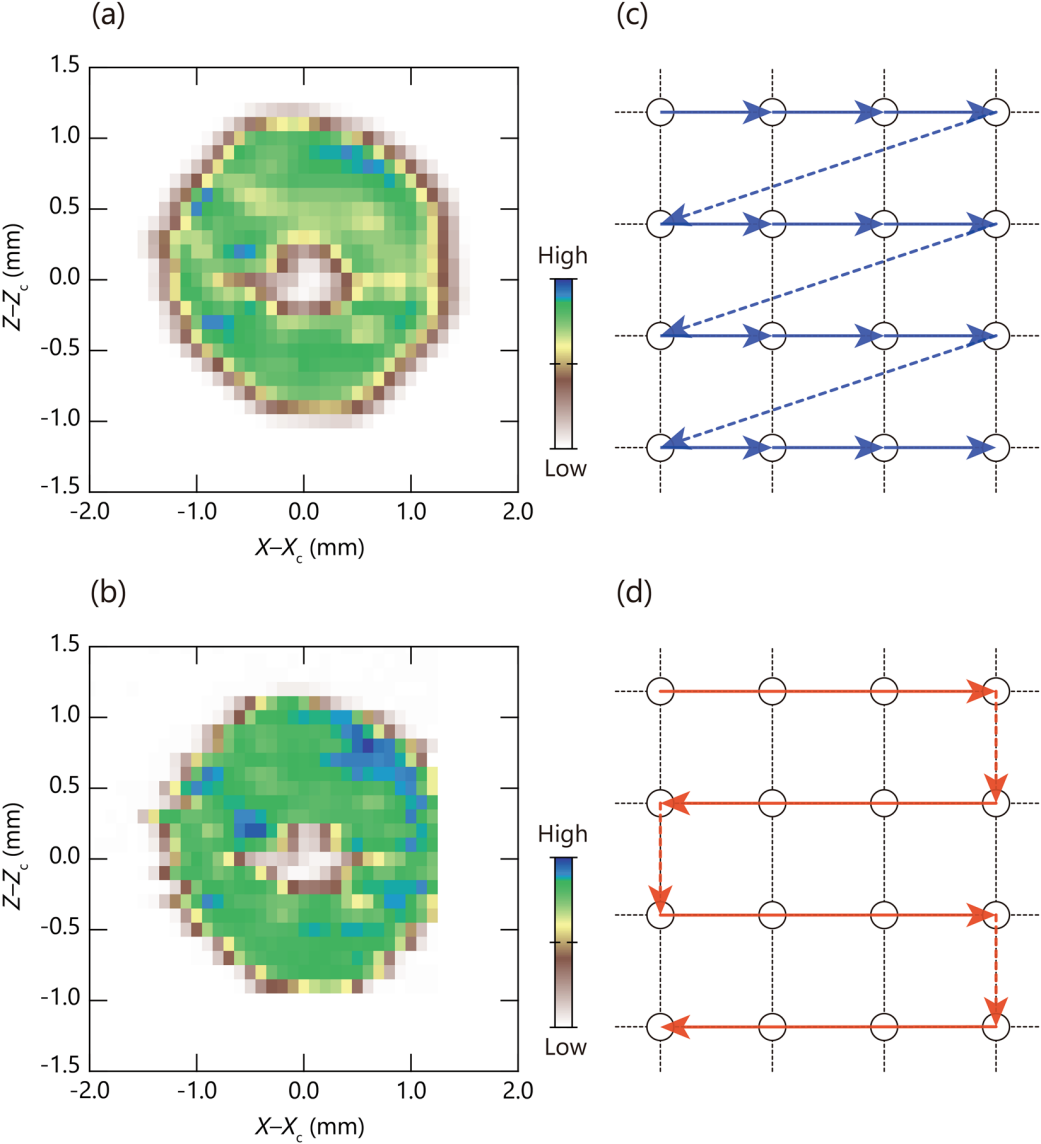
Evaluation of scanning acquisition method via spatial imaging measured from a high-*T*_c_ superconducting cuprate Bi_2_Sr_2_CaCu_2_O_8+δ_ taken with a photon energy of 6.43 eV and a temperature below 20 K near the nodal direction. (**a**,**b)** Two dimensional spatial imaging of ARPES intensity of Bi2212 by (**a**) the stepping method and (**b**) the scanning method, where the full integration windows in energy and momentum were employed to the nodal ARPES spectra similar to Fig. 3(d4). (**c**,**d)** Schematic drawings illustrate the motion control in the stepping and scanning method, respectively. Each arrow represents the motion control in one action. Note that unidirectional and bidirectional acquisitions were also employed as indicated by the solid lines. The ARPES acquisitions have been stopped in the regions indicated by the dashed lines. In the scanning mode, the sample positions were finely tuned at both arrow edges several times by reflecting feedbacks of the encoder to reduce errors due to backlash.

The scanning ARPES acquisition method was examined via a two-dimensional spatial imaging of Bi2212 taken again with 6.43 eV below 20 K. Figure 5a,b show a two-dimensional scanning photoemission microscopy (SPEM) image of Bi2212 measured by the (a) stepping and (b) scanning methods, where the intensity of each pixel was obtained from the nodal APRES spectra of Bi2212 similar to Fig. 3(d4) by integrating over the full energy and momentum windows. As shown in Fig. 5c,d, the motion control was done unidirectionally in the stepping method to ensure the accuracy of coordinate information, while it becomes bidirectionally in the scanning method to put importance on the acquisition speed. In addition, we used the so-called snapshot (or fixed) mode in which the photoemission spectrum can be obtained in a single shot from the image on the two-dimensional detector. Hence, the dwell-time basically corresponds to the acquisition time per spectrum except for the overhead time. It should be noted that the fixed-mode acquisition holds a disadvantage that the photoemission intensity should reflect detector inhomogeneity, though such effects can be eliminated in the constructed SPEM image by integrating over an energy window.

Each pixel of the SPEM image in the stepping method shown in Fig. 5a was measured with 100 μm step size both in the *X* and *Z* axes, both of which were rescaled by the center of the sample (*X*_c_ and *Z*_c_). Here, a total of 858 data points were mapped out with an acquisition time of 126 min. 48 sec in total, meaning 8.87 sec per step. As we used the dwell time of 4 sec per spectrum, an overhead time was estimated to be 4.87 sec per step and total overhead time of the whole map was 69 min. 38 sec. The results indicate ~ 55% of total acquisitions time was overhead time.

In the scanning ARPES acquisition method, the number of data points are determined by the dwell-time per spectrum and the motor velocity. We thus used the same dwell time of 4 sec per spectrum, but adjusted the motor velocity as to have a similar number of data points compared to the stepping acquisition measurement. As a result, the SPEM image measured by the scanning acquisition method shown in Fig. 5b has a similar 832 data points with same 100 *μ*m steps in both *X* and *Z* axes. These results were obtained with a total acquisition time of 60 min. 20 sec, and thus, the acquisition time of single spectrum was 4.35 sec, giving an estimate of overhead time of 0.35 sec per one spectrum, and hence, total overhead time of 4 min. 52 sec, which is only ~ 8% of the total acquisition time.

It should be emphasized here that the obtained intensity distributions shown in Fig. 5a,b are essentially identical between two methods. Nevertheless, the required data acquisition time as well as overhead time were drastically reduced in the scanning acquisition method by ~ 50%. This demonstrates the effectiveness of the scanning ARPES acquisition method in the high-resolution ARPES microscopy experiments. This accelerated data acquisition method would be also helpful to reduce possible radiation damages on the sample increased with the smaller focused beam size as reported in functional oxide materials^11,12^.

## Conclusion

In summary, we presented two approaches to advance high-resolution ARPES microscopy experiments. Rotational displacements can be compensated by tracing ARPES acquisition method in which new coordinates are successively predicted from the target positions based on the proper center of rotation. We demonstrated via the Fermi surface mapping of Bi2212 that the tracing ARPES method enabled more accurate μ-ARPES experiments to prevent a contamination of the signals from different surface domains by keeping the measurement position on the sample surface during angular rotations. We also demonstrated that scanning ARPES acquisition method can drastically reduce total data acquisition time by significantly eliminating overhead time. We believe that present ARPES acquisition methods will significantly help improving the accuracy, efficiency, and throughput of high-resolution ARPES microscopy experiments, especially when requiring high-accuracy or treating a large number of data points. Our methods developed herein would be of great significance for further studies of intrinsic local electronic structures of functional materials in micro scales, and are thus anticipated to be beneficial for developments of micro-scale electronic devices and their applications.

## Methods

### μ-ARPES experiments and materials

All the experiments were performed using a newly developed laser-based μ-ARPES at the Hiroshima Synchrotron Radiation Center (HiSOR) with high energy-, angular-, and spatial-resolutions (<260 *μ*eV, <0.05°, and <5 *μ*m). Present μ-ARPES system equipped with a high-resolution hemispherical electron analyzer (R4000, Scienta Omicron), a tunable (5.90–6.49 eV) vacuum ultraviolet (VUV) laser consisting of a fourth harmonic generator (HarmoniXX, A·P·E) and a mode-locked Ti:sapphire laser (Millenia eV, Spectra Physics), a high precision translator *XYZ* stage (iXYZ, the ExPP Co. Ltd.) with differentially pumped rotational stage (iRS152, Vacuum and Optical Instruments Co. Ltd.)^8^, and a two-axis sample goniometer with a liquid-helium-flow cryostat (*i*-GONIO LT, R-Dec Co. Ltd.)^7^. Details of the μ-ARPES system at HiSOR are described in the literature^4^. Present μ-ARPES data were measured at the photon energy of 6.43 eV and a temperature below 20 K on optimally doped Bi2212 (*T*_c_ = 92 K). High-quality single crystals of optimally doped Bi2212 were prepared by the traveling-solvent floating-zone technique^13^. A clean surface of the samples was obtained by cleaving *in situ* in ultrahigh vacuum better than 4 × 10^−11^ Torr below 20 K. The energy resolution was set to be better than 5 meV, and angular resolution was better than 0.05 deg.

## Electronic supplementary material

Supplementary Material

## Data Availability

The data and codes that support the findings of this study are available from the corresponding author upon reasonable request.
