# Supplementary material for: Accurate and efficient data acquisition methods for high-resolution angle-resolved photoemission microscopy
Source: Sci Rep. 2018 Nov 27;8:17431. doi: 10.1038/s41598-018-34894-7 (PMC6258666; doi:10.1038/s41598-018-34894-7)
Supplement: Supplementary file 1 — Supplementary Material [file 41598_2018_34894_MOESM1_ESM.pdf]

Supplementary Material: Accurate and efficient data acquisition methods for high-resolution angle-resolved photoemission microscopy

Hideaki Iwasawa[1,2,3], Hitoshi Takita[3], Kazuki Goto[3], Wumiti Mansuer[3], Takeo Miyashita[3], Eike F. Schwier[1], Akihiro Ino[1,4], Kenya Shimada[1], Yoshihiro Aiura[5]

[1]*Hiroshima Synchrotron Radiation Center, Hiroshima University, Higashi-Hiroshima, Hiroshima 739-0046, Japan*
[2]*Diamond Light Source, Harwell Science and Innovation Campus, Didcot OX11 0DE, UK*
[3]*Graduate School of Science, Hiroshima University, Higashi-Hiroshima, Hiroshima 739-8526, Japan*
[4]*Faculty of Engineering, Kurume Institute of Technology, Kurume, Fukuoka 830-0052, Japan*
[5]*National Institute of Advanced Industrial Science and Technology, Ibaraki 305-8568, Japan*
*Correspondence and requests for materials should be addressed to H.I. (email: h-iwasawa@hiroshima-u.ac.jp).

Supplementary Note: Determination of the center of rotation

Here we present the determination of the center of rotation that is essential to calculate the trajectory of a target position by a 4x4 matrix as described in Eq. 1. In our developed μ-ARPES system [S1], the tilt rotation is used for the Fermi surface mapping as the direction of the analyser slit is horizontal, and the tilt (ϕ) and polar (ϑ) rotations are thus rather important in terms of in-plane *k*-space mapping than the azimuthal rotation (φ). Hence, we have examined the trajectory of an incident laser spot on a phosphor sample using long distance microscopes while only giving a polar or tilt rotation of the sample with a constant azimuthal rotation (φ=0). Figures S1(a) shows the experimental geometries, where two microscope cameras (Camera1 and Camera2) were installed; Camera1 is located diagonally 45° upward from the laser path for monitoring the polar rotation, while Camera2 is placed in the *XY*-plane and at the 45° between the *X*- and *Y*-axes.

Figures S1(b1)-(b3) show the optical images taken with Camera1 at (b1) an original position with ϑ = 0° ($\phi = \varphi$ = 0°), at (b2) ϑ = +5° ($\phi = \varphi$ = 0°), and (b3) ϑ = +5° ($\phi = \varphi$ = 0°) after compensating rotational displacements by translating *X* and *Y* coordinates. Figures S1(c1)-(c3) correspond to magnified images as indicated by the yellow region in Figs. S1(b1)-(b3). The laser spot can be clearly identified in Figs. S1(b1,c1) with the sample position of (X_0,Y_0). While the spot was not visible after the polar rotation in Figs. S1(b2,c2), it could be recovered to the original position on the sample with new coordinates (X_1,Y_1) in Figs. S1(b3,c3). This means that the polar rotation of ϑ = +5° transform (X_0,Y_0) to (X_1,Y_1). By performing such procedures at each polar angle, we have manually traced the trajectory of the laser spot as a function of the polar angle as plotted in Fig. S1(f). The obtained trajectory was well-fitted by Eq. 1, resulting in the rotational center of (X_{CR}, Y_{CR})=(42.662, 37.034) in mm unit. Similarly, the results for the tilt rotation (ϕ = +13°) are presented in Figs. S1(d1)-(d3) and S1(e1)-(e3). The tilt rotation slightly moved the spot on the sample as seen in Figs. S1(d1,e1) and S1(d2,e2), and the *Y* and *Z* translation successfully compensated the rotational displacements as shown in Figs. S1(d3,e3). Figure S1(g) summarizes the trajectory of the laser spot on the sample as

a function of the tilt angle, which can be well reproduced by fitting with (Y_{CR}, Z_{CR})=(38.035, 127.100) in mm unit.

We found a significant deviation (~1 mm) on the Y_{CR} between two examination results obtained by polar and tilt rotations. These results can be understood, if the *XYZ* rotation axes actually do not meet at a specific point in the present system, as reasonably expected in a real experimental system. Consequently, for the proper calculation of the three dimensional rotation, we have modified Eq. 1 using the center of rotation for each rotations as follows;

$$\begin{pmatrix} x_1 \\ y_1 \\ z_1 \\ 1 \end{pmatrix} = T_{\mathrm{CR}}^{+\vartheta} R_Z(\vartheta) T_{\mathrm{CR}}^{-\vartheta} T_{\mathrm{CR}}^{+\varphi} R_Y(\varphi) T_{\mathrm{CR}}^{-\varphi} T_{\mathrm{CR}}^{+\phi} R_X(\phi) T_{\mathrm{CR}}^{-\phi} \begin{pmatrix} x_0 \\ y_0 \\ z_0 \\ 1 \end{pmatrix}, \quad \text{(S1)}$$

where $T_{\mathrm{CR}}^{\pm\phi}, T_{\mathrm{CR}}^{\pm\varphi}, T_{\mathrm{CR}}^{\pm\vartheta}$ are the translational matrix given by

$$T_{\mathrm{CR}}^{\pm\phi} = \begin{pmatrix} 1 & 0 & 0 & \pm x_{\mathrm{CR}}^{\phi} \\ 0 & 1 & 0 & \pm y_{\mathrm{CR}}^{\phi} \\ 0 & 0 & 1 & \pm z_{\mathrm{CR}}^{\phi} \\ 0 & 0 & 0 & 1 \end{pmatrix}, T_{\mathrm{CR}}^{\pm\varphi} = \begin{pmatrix} 1 & 0 & 0 & \pm x_{\mathrm{CR}}^{\varphi} \\ 0 & 1 & 0 & \pm y_{\mathrm{CR}}^{\varphi} \\ 0 & 0 & 1 & \pm z_{\mathrm{CR}}^{\varphi} \\ 0 & 0 & 0 & 1 \end{pmatrix}, T_{\mathrm{CR}}^{\pm\vartheta} = \begin{pmatrix} 1 & 0 & 0 & \pm x_{\mathrm{CR}}^{\vartheta} \\ 0 & 1 & 0 & \pm y_{\mathrm{CR}}^{\vartheta} \\ 0 & 0 & 1 & \pm z_{\mathrm{CR}}^{\vartheta} \\ 0 & 0 & 0 & 1 \end{pmatrix},$$

where $\pm x_{\mathrm{CR}}^{\phi}, \pm y_{\mathrm{CR}}^{\varphi}, \pm z_{\mathrm{CR}}^{\vartheta}$ are indeed negligible parameters because each rotation is done along the same axis. The rotational matrix with respect to the *X*, *Y*, *Z*-axis, $R_X(\phi)$, $R_Y(\varphi)$, $R_Z(\vartheta)$, are same as in Eq. 1 and respectively given by

$$R_X(\phi) = \begin{pmatrix} 1 & 0 & 0 & 0 \\ 0 & \cos\phi & \sin\phi & 0 \\ 0 & -\sin\phi & \cos\phi & 0 \\ 0 & 0 & 0 & 1 \end{pmatrix}, R_Y(\varphi) = \begin{pmatrix} \cos\varphi & 0 & -\sin\varphi & 0 \\ 0 & 1 & 0 & 0 \\ \sin\varphi & 0 & \cos\varphi & 0 \\ 0 & 0 & 0 & 1 \end{pmatrix},$$

$$R_Z(\vartheta) = \begin{pmatrix} \cos\vartheta & \sin\vartheta & 0 & 0 \\ -\sin\vartheta & \cos\vartheta & 0 & 0 \\ 0 & 0 & 1 & 0 \\ 0 & 0 & 0 & 1 \end{pmatrix}.$$

The modified Eq. S1 was employed for evaluating the tracing acquisition method as shown in Fig. 3. It should be also noted that a transfer from a reference sample to real samples requires a precise control of external forces on the manipulator and cryostat to reproduce the same center of rotation each time.

Supplementary Reference

[S1] Iwasawa, H. *et al.* Development of laser-based scanning μ-ARPES system with ultimate energy and momentum resolutions. *Ultramicroscopy* **182**, 85–91 (2017).

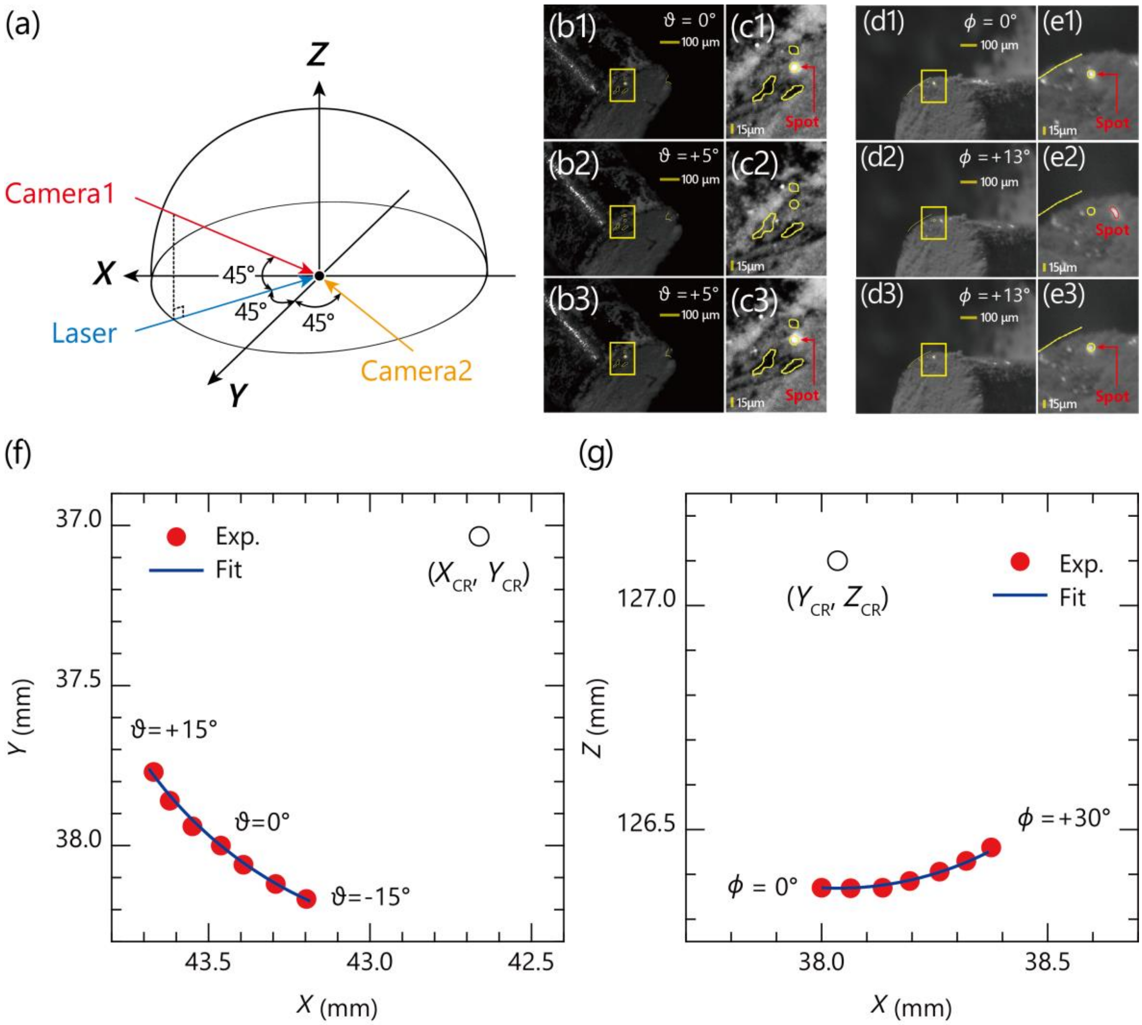

Fig. S1 Determination of the center of rotation by manually tracing the laser spot on a phosphor sample while giving polar or tilt rotations of the sample. **a** Schematic experimental geometry, indicating the positions of the microscope cameras (Camera1 and Camera2). The Camera1 was installed from diagonally upward 45 deg by the laser path for monitoring a polar rotation, while the Camera2 was installed in the *XY*-plane and from the 45 deg by the *X* and *Y* axes for monitoring a tilt rotation. **b1-3,d1-3** Optical microscope images taken by the Camera1 and Camera2, respectively, where the images were taken at (b1, d1) an original position, (b2, d2) at $\vartheta = +5°$ and $\phi = +13°$, respectively, without translational corrections, and (b3, d3) same with (b2, d2) but including translational corrections. **c1-3,e1-3** Magnified images as indicated of yellow rectangles in (b1-b3) and (d1-d3), respectively. The yellow markers in all the images placed at fixed pixel positions. **f,g** Experimentally determined trajectory of the laser spot on the sample (red lines) for polar and tilt rotations, respectively, which were reproduced by the calculations (blue lines) assuming the center of rotations (open circles).
